# On the evolution of cellular senescence

**DOI:** 10.1111/acel.13270

**Published:** 2020-11-09

**Authors:** Axel Kowald, João F. Passos, Thomas B. L. Kirkwood

**Affiliations:** ^1^ Campus for Ageing and Vitality Newcastle University Institute for Ageing Newcastle upon Tyne UK; ^2^ Rostock University Medical Center Institute for Biostatistics and Informatics in Medicine and Aging Research (IBIMA) Rostock Germany; ^3^ Department of Physiology and Biomedical Engineering, Robert and Arlene Kogod Center on Aging Mayo Clinic Rochester Minnesota USA; ^4^ Center for Healthy Aging Department of Cellular and Molecular Medicine University of Copenhagen Copenhagen Denmark

**Keywords:** aging, anti‐aging, cellular senescence, evolution, senolytics

## Abstract

The idea that senescent cells are causally involved in aging has gained strong support from findings that the removal of such cells alleviates many age‐related diseases and extends the life span of mice. While efforts proceed to make therapeutic use of such discoveries, it is important to ask what evolutionary forces might have been behind the emergence of cellular senescence, in order better to understand the biology that we might seek to alter. Cellular senescence is often regarded as an anti‐cancer mechanism, since it limits the division potential of cells. However, many studies have shown that senescent cells often also have carcinogenic properties. This is difficult to reconcile with the simple idea of an anti‐cancer mechanism. Furthermore, other studies have shown that cellular senescence is involved in wound healing and tissue repair. Here, we bring these findings and ideas together and discuss the possibility that these functions might be the main reason for the evolution of cellular senescence. Furthermore, we discuss the idea that senescent cells might accumulate with age because the immune system had to strike a balance between false negatives (overlooking some senescent cells) and false positives (destroying healthy body cells).

## Graphical abstract

Cellular senescence is often regarded as an anti‐cancer mechanism although many studies have shown that senescent cells often also have carcinogenic properties. Here, we discuss the possibility that actually wound healing, tissue repair, and development might be the main reason for the evolution of cellular senescence. We also suggest that senescent cells might accumulate with age because the immune system has to strike a balance between false negatives and false positives.

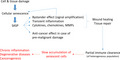

## 1 INTRODUCTION

It is 60 years since the phenomenon of cell replicative senescence was discovered in human diploid fibroblasts (Hayflick & Moorhead, 1961). At that time, the accepted view was that cultured cells would grow indefinitely if provided with suitable conditions. After the validity of the new discovery was accepted, it was shown that an important difference existed between “normal” cells, which have finite replicative life spans, and malignantly “transformed” cells, which are able to proliferate indefinitely. Although poorly understood, senescence was suggested to be evidence of intrinsic aging occurring at the cellular level. This was supported by reports that cell replicative life span (expressed as the number of population doublings) was correlated with (a) the longevity of the species from which cultures were grown (Röhme, 1981), and (b) the age of the donor from which the biopsies were obtained (Martin et al., 1970). Although a simple relationship between organismal aging and the idea of cells merely running out of division potential came to be questioned (Cristofalo et al., 1998, 2004), the idea of a causal connection persisted, despite the fact that such a connection could not yet be shown in vivo. An important advance was therefore the discovery of markers, such as senescence‐associated β‐galactosidase (SA‐β‐gal) and p16 (Campisi & d'Adda di Fagagna, 2007; Collins & Sedivy, 2003; Dimri et al., 1995). These were used not only to identify senescent cells within tissues, but also to show that they increase with age in vivo (Burd et al., 2013; Yamakoshi et al., 2009). Studies designed to look at different tissues found values between 2%–14% in old mice (Biran et al., 2017). However, none of the current markers identify senescent cells unequivocally, so senescence is probably best determined by a combination of multiple markers until its identification has been further resolved.

The next key step was the discovery that replicative senescence could be caused by the erosion of telomeres—the protective structures capping the ends of linear chromosomes (Harley et al., 1990). Telomere erosion occurs because of the inability of DNA polymerases to copy the very ends of the chromosomes. In germ cells and certain other specialized cell types, this limitation is overcome by the actions of telomerase, but in fibroblasts and many other differentiated cell types, telomerase expression is switched off. This suggested initially that senescence might be a programmed process in which the telomeres acted as a form of molecular clock. Against the idea of a simple clock, however, was the finding that replicative senescence exhibits marked heterogeneity in the division potential of the individual cells within the population, and even in clonally derived sub‐populations (Smith & Whitney, 1980). Furthermore, evolutionary considerations argued not only against aging being programmed but also against the idea of it having a single molecular cause (Kirkwood, 2005). Theoretical modeling of the interactions between different candidate mechanisms of molecular aging (somatic mutations, mitochondrial dysfunction, telomere erosion) indicated that the observed heterogeneity in cell division potentials could be explained by the action of multiple mechanisms acting together (Sozou & Kirkwood, 2001). This led to experimental tests of this possibility, which revealed that the random effects of mitochondrial mutations (resulting in intracellular oxidative stresses, to which telomeres are particularly susceptible) could account for the stochastic heterogeneity in telomere‐driven replicative senescence (Passos et al., 2007). At the same time, it was found that not only telomere attrition but also a diverse range of damaging conditions (oxidative stress, DNA damage, radiation, or the expression of certain oncogenes), all of which involve DNA damage in some form, could trigger cellular senescence (CS) (Campisi, 2013; Coppe et al., 2010; Gorgoulis et al., 2019).

In response to the evidence that pathways leading to establishment of senescence were proving to be more complex than previously envisaged, efforts were made to combine the power of bioinformatics and systems modeling with functional analysis of gene regulation. This revealed that there exists a dynamic feedback loop that is triggered by a DNA damage response (DDR) and which, after a delay of several days, locks the cell into an actively maintained state of “deep” cellular senescence (Passos et al., 2010). The essential feature of this discovery was that cellular senescence was a regulated process offering an alternative response to damage than the option of cellular “suicide,” known as apoptosis. Although apoptosis provided a means to remove a damaged cell completely, senescence allowed the cell to remain but permanently removed its potential for further division. Furthermore, many senescent cells are highly resistant to the induction of apoptosis (Childs et al., 2014).

At first sight, senescence and apoptosis could simply be seen as complementary alternatives to managing the potentially harmful effects of acquired cellular damage (Childs et al., 2014), especially with respect to the risk of cancer. If the cell type is high‐risk, such as a stem cell, apoptosis would get rid of it altogether. However, there was some indication that boosting apoptosis resulted in faster aging by accelerating the age‐related loss in tissue cellularity (Kirkwood, 2002; Tyner et al., 2002). It was conceivable, therefore, that there might be circumstances in which the damaged cell would better be preserved, while being locked out of the possibility of further division. But of course things are never as straightforward as they seem at first sight. It was already clear that apoptosis had more roles than protection against cancer, since it is essential, for example, during morphogenesis and in managing the risk of autoimmune reactions during hematopoiesis. With cellular senescence, an important discovery was that most senescent cells undergo alteration to produce the “senescence‐associated secretory phenotype” (SASP) (Coppe et al., 2010; de Keizer, 2017). The SASP involves the production of a complex array of chemokines, cytokines, growth factors, and proteases, which cause significant effects on neighboring cells, even including conversion into new senescent cells by way of the so‐called “bystander” effect (Nelson et al., 2012; da Silva et al., 2018; Xu et al., 2017). Many of the impacts of the SASP appear to be negative: it promotes chronic inflammation, which in turn is an important contributor to a wide range of age‐related diseases. However, as with apoptosis, senescent cells turn out to have beneficial effects in development, wound healing, and tissue repair (Demaria et al., 2015; Gal et al., 2019; Gibaja et al., 2019; Ritschka et al., 2017).

A turning point in perception of senescent cells and their relationship to aging and health was the finding in mice that the targeted removal of senescent cells, termed “senolysis,” resulted in increased life span and beneficial effects on health (Baar et al., 2017; Baker et al., 2011, 2016; de Keizer, 2017; Ovadya et al., 2018; Xu et al., 2018). However, cellular senescence is a complex phenomenon that is far from being fully understood as indicated by recent findings that the removal of non‐replaceable senescent cells in the liver actually shortens the life span of mice (Grosse et al., 2020). Nevertheless, major efforts are now underway to examine whether similar approaches might deliver improvements to health during human aging.

Given the complexity of what is known already about cellular senescence, it seems prudent to consider why and how natural selection might have shaped the roles of senescent cells in our bodies, in the hope that this might also deliver new insights into future therapeutic possibilities.

## 2 EVOLUTION AND AGING

When considering why and how features of aging might have evolved, the first thing to appreciate is that evolutionary logic does not support the idea that aging itself is due to a genetic program (Kirkwood & Melov, 2011). Although the appeal of programmed aging is understandable, aging cannot be explained easily this way, if at all (Kowald & Kirkwood, 2016). Biological old age is rarely attained in natural populations, and it therefore makes little sense to expect that evolution resulted in a process that is seldom seen. Furthermore, aging is deleterious to the individual, and natural selection should oppose rather than promote it. The body is programmed for survival, not death. But because survival to high ages is rare in natural populations, there would not have been any evolutionary pressure to maintain the body well enough to last forever (Kirkwood, 1977; Kirkwood & Holliday, 1979). This conclusion, embodied in the “disposable soma” theory, is that aging results from progressive accumulation of molecular and cellular damage due to evolved limitations in maintenance and repair. The same logic explains how in different species, where the exposure to natural hazards is different, the limitations on maintenance and repair would be tuned accordingly. This is confirmed by evidence that cells from longer‐lived species are generally better protected than cells from shorter‐lived species (Kapahi et al., 1999; Ma et al., 2016). It also explains why the age‐incidence curves of damage‐related diseases, such as cancer, scale with life span.

The fact that aging is not programmed in itself does not, however, exclude the possibility that secondary consequences of the aging phenotype are the result of evolutionary programming. Damage is a ubiquitous threat to all living systems, and it is only to be expected that adaptations to deal with damage are fundamental. In multicellular organisms, the risk to the organism that arises from damage to individual cells is countered by regulated responses, in particular, apoptosis and cellular senescence. The fundamental nature of cellular senescence as damage response is also highlighted by the fact that associated genes are more highly conserved in mammals than would be expected by chance (Avelar et al., 2020). Damage also arises through wounding and infections, for which protective responses are provided by immune and inflammatory mechanisms. Although much interest currently focuses on the consequences that responses such as senescence, inflammation, and apoptosis may have for health at older ages, it is important to appreciate that the origin of these responses needs to be sought in the benefit they confer at younger ages. The idea that evolution might have produced a trait that is good in youth but harmful in later life is known as “antagonistic pleiotropy” (Rose & Graves, 1989; Williams, 1957).

As we proceed to address the way that natural selection may have shaped the roles of cellular senescence, both of the above concepts—disposable soma and antagonistic pleiotropy—will be relevant. The concepts are complementary, not exclusive.

## 3 CELLULAR SENESCENCE AS ANTI‐CANCER STRATEGY

**Figure 1:**
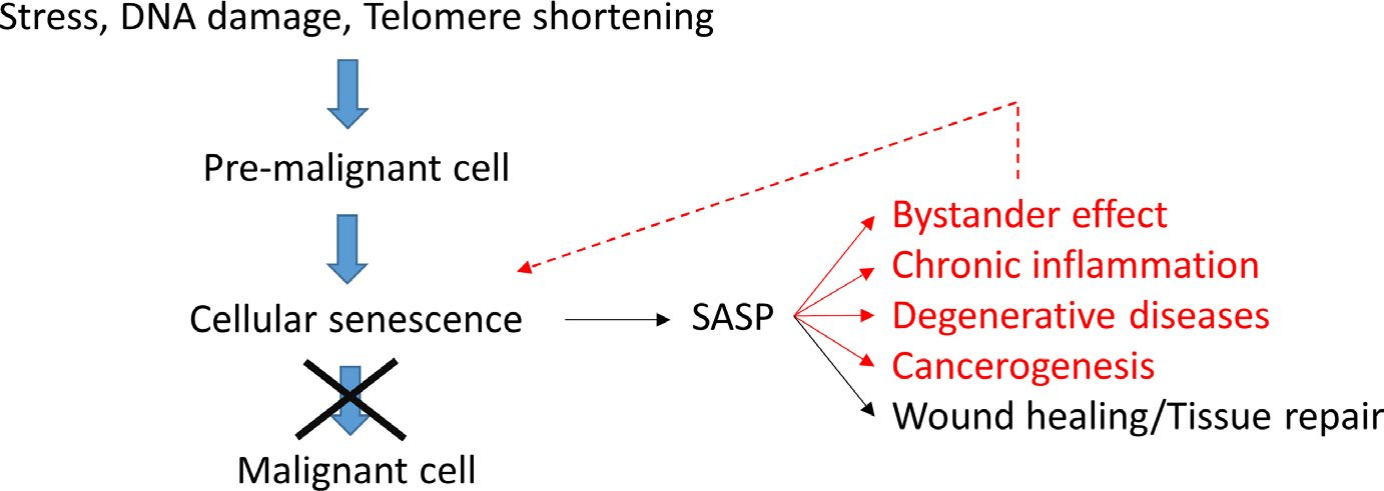
Cellular senescence has been suggested to be an anti‐cancer strategy. However, the senescence‐associated secretory phenotype (SASP) has many negative consequences (shown in red), which are difficult to reconcile with this idea

What might be the “purpose” of cellular senescence? More accurately phrased, what could be the selective advantage that led to its evolution in so many species? The most popular idea today is that cellular senescence is a mechanism that helps to suppress the development of cancer (Sager, 1991). This idea has also been suggested by several others (Campisi, 2013; Campisi & d'Adda di Fagagna, 2007; Coppe et al., 2010) (and refs within) and is conceptually visualized by Figure 1. According to this proposal, different types of stress and damage can lead to the generation of pre‐malignant cells. Cellular senescence is then the mechanism that senses this state and prevents further progression into a full‐blown malignant state by permanently withdrawing the cell from the cell cycle.

However, senescent cells have a property that is hard to reconcile with this picture. They display a senescence‐associated secretory phenotype (SASP), which consists of a complex cocktail of chemokines, cytokines, growth factors, and proteases (Coppe et al., 2010; de Keizer, 2017). The SASP can have a large range of effects, most of which are negative for organismal health (Figure 1). The bystander effect, for instance, describes the fact that the paracrine action of the SASP can convert neighboring cells into new senescent cells (Acosta et al., 2013; Nelson et al., 2012; da Silva et al., 2018; Xu et al., 2017), thus amplifying and spreading the original generation of senescent cells to affect non‐damaged, healthy cells. Although the composition of the SASP is somewhat heterogeneous (Coppe et al., 2010), a general property is that it promotes inflammation (Campisi, 2013; Hernandez‐Segura et al., 2018). Chronic inflammation in turn is an important contributor to many age‐related diseases and it has been shown that senescent cells, directly or indirectly, are causally involved in diseases such as atherosclerosis, fibrosis, pancreatitis, osteoarthritis, Alzheimer disease, and metabolic disorders (Pignolo et al., 2020). But perhaps most surprisingly, senescent cells (via SASP) are also involved in carcinogenesis and hyperplasic pathology (Campisi, 2013; Gonzalez‐Meljem et al., 2018; Wang et al., 2017; Yanai & Fraifeld, 2018). It has been known for some time that acute wounds accelerate the growth of tumors in their neighborhood (Stuelten et al., 2008). Considering that senescent cells are involved in wound healing (see next section), this is a further indication of the pro‐tumorigenic properties of SASP and senescent cells.

How can a process that initiates or promotes cancer at the same time evolve as anti‐cancer strategy? The explanation put forward is antagonistic pleiotropy (Campisi, 2013; Campisi & d'Adda di Fagagna, 2007; Coppe et al., 2010). If a mechanism has beneficial effects early in life, but negative effects late in life, evolution theory predicts that, because the force of natural selection declines with age, such a trait can have an overall selection advantage (Williams, 1957). Thus, if cellular senescence prevents cancer in young animals, but at a later age also promotes cancer, the early benefits might outweigh the later adverse effects, such that senescence can nevertheless evolve.

Evolution theory would also predict, however, that over evolutionary time the link between the beneficial and detrimental effects is broken, if possible. That means if the negative effects that emerge as a consequence of the SASP can be separated (i.e., eliminated) from the positive, anti‐tumorigenic effects of cellular senescence, then we would expect to see this happen since it would increase overall fitness. An obvious way to achieve this would simply be if senescent cells would not have the associated secretory phenotype. True, in this case, the beneficial effects of SASP on wound healing and tissue repair would also be affected, but this function could be delegated to other cell types. Similarly, the autocrine reinforcement of the senescent state, that seems to be mediated by some SASP components (Acosta et al., 2008; Campisi, 2013; Hinds & Pietruska, 2017; Kuilman et al., 2008), could also be converted into a purely intracellular signaling pathway.

Furthermore, there is an even more radical way to avoid the negative effects of senescent cells and SASP while still providing an anti‐cancer mechanism. That alternative is, of course, apoptosis. If a cell has suffered damage that is beyond repair, a cell can trigger a suicide program that results in the removal from the body without causing any inflammation. Apoptosis is an effective anti‐cancer mechanism and its deregulation is involved in many types of cancer (Pistritto et al., 2016). Not only does apoptosis avoid the negative consequences of the SASP, but it also completely removes potentially pre‐malignant cells, instead of only rendering them post‐mitotic. Apoptosis thus seems to be an anti‐cancer strategy with much fewer problems than cellular senescence.

Moreover, a recent analysis of 279 human genes involved in cellular senescence showed that genes inducing cellular senescence statistically overlapped with anti‐longevity genes and not with pro‐longevity genes (Avelar et al., 2020). The same study also demonstrated that there is a significant overlap of oncogenes with inducers as well as inhibitors of senescence. This is not what would be expected from a life‐extending anti‐tumor mechanism.

## 4 CELLULAR SENESCENCE AS TISSUE REPAIR & REMODELING MECHANISM

As explained in the last section, the idea that cellular senescence evolved as anti‐cancer strategy has logical problems and inconsistencies. Here, we now present an alternative view of the evolution of cellular senescence, which readily explains many of the experimental observations and which is consistent with evolution theory. In principle the idea is based on the finding that the senescence‐associated secretory phenotype (SASP) is important for several biological processes that are not related to aging, such as limiting liver fibrosis (Krizhanovsky et al., 2008), accelerating and improving wound healing (Demaria et al., 2014, 2015), tissue regeneration (Ritschka et al., 2017) and limb regeneration of salamanders (Yun et al., 2015). Furthermore, senescent cells are also found during embryogenesis in the apical ectodermal ridge and the neural roof plate (Storer et al., 2013), during development of the placenta (Chuprin et al., 2013; Gal et al., 2019; Rajagopalan & Long, 2012) as well as during the development of the inner ear (Gibaja et al., 2019; Munoz‐Espin et al., 2013) (for a recent review see also Rhinn et al., (2019)). But instead of regarding these observations as a side effect, they can be seen as the starting point to provide a different explanation for the evolution of cellular senescence.

As previously observed by others, senescent cells are involved in development as well as tissue and wound healing and we suggest that this is the driving force behind their evolution. The term “senescent cell” might thus be quite misleading and distracts from the main task of this special physiological cell state. Senescent cells are created not only via telomere attrition, but also by many damage and stress factors like reactive oxygen species, radiation, or chemotherapy (Campisi, 2013; Coppe et al., 2010). Such stress factors could be created directly during tissue damage or might be produced as a consequence of such damage. In any case, senescent cells are created at the location of tissue damage and remodeling and support the healing process via their secretory phenotype. The SASP causes an inflammation, attracts immune cells, and makes it easy for immune cells to get to the problem area via its matrix metalloproteases. In this scenario, the SASP is not detrimental, but serves a specific purpose. Consequently, it also makes sense that senescent cells are resistant to apoptosis, since they have to be present until the healing process is completed. Furthermore, it would be useful if senescent cells can turn normal cells in their neighborhood into senescent cells as a way to amplify the healing signal. This can explain the observed bystander effect of senescent cells (Acosta et al., 2013; Nelson et al., 2012; da Silva et al., 2018; Xu et al., 2017). After wound healing/tissue remodeling is completed, senescent cells are removed. This is normally performed by the immune system and it seems that various cell types from macrophages and natural killer cells to cytotoxic T cells are involved in the process (Burton & Stolzing, 2018; Kale et al., 2020; Yun et al., 2015). Indeed, immunocompromised mice that have defective cytotoxic T cells develop chronic inflammation, accumulate senescent cells much faster and die 20% earlier (Ovadya et al., 2018).

The evolutionary roots of apoptosis and cellular senescence reach deep. It has been proposed that apoptosis in eukaryotes is connected to the endosymbiotic origin of mitochondria (Blackstone & Kirkwood, 2003), but programmed cell death can also be found in various bacteria to increase the fitness of the colony in response to adverse conditions (Allocati et al., 2015). Similarly, cellular senescence in the form of a limited division potential can also be traced back to unicellular organisms like yeast (Jazwinski, 1990) and bacteria (Stewart et al., 2005). According to our view, both mechanisms fulfill important and complementary functions in the adult organism (removal of different types of damage) as well as during development (see above). The profound role of senescent cells during tissue remodeling and regeneration is supported by findings that transient exposure to SASP induces de‐ and trans‐differentiation in primary mouse keratinocytes (Ritschka et al., 2017). Together with the important role of senescent cells during salamander limb regeneration (Yun et al., 2015) (which also involves dedifferentiation) this reinforces the idea that the primary role of cellular senescence lies with damage repair and tissue patterning.

## 5 SENESCENCE IN POST‐MITOTIC CELLS

An unexpected recent finding is that post‐mitotic cells (such as cardiomyocytes, neurons, adipocytes, retinal ganglion cells, osteocytes, and osteoblasts) can acquire a multitude of senescent markers during aging (Farr et al., 2016; Jurk et al., 2012; Minamino et al., 2009; Oubaha et al., 2016; Anderson et al., 2019). Importantly, it has been shown that elimination of senescent post‐mitotic cells is also accompanied by beneficial effects in these tissues (Anderson et al., 2019; Farr et al., 2017; Ogrodnik et al., 2019). Senescent post‐mitotic cells have been shown to express genes such as p21 and p16 involved in cell‐cycle arrest and have a SASP (Anderson et al., 2019; Farr et al., 2017; Jurk et al., 2012). In the case of post‐mitotic senescence, we are referring to cells which are terminally differentiated (already exited from the cell cycle) but that experience exacerbation of several markers of senescence during aging. Existing evidence suggests that this process is the result of random molecular damage and the fact that one sees an age‐dependent increase suggests that the pathways mediating terminal differentiation are quite distinct.

The mechanisms underlying senescence in post‐mitotic cells are less clear, however, it has been suggested that, similarly to the situation in proliferation‐competent cells, mitochondrial dysfunction and oxidative damage to telomere regions may be driving factors (Anderson et al., 2019). From the evolutionary perspective, it is not obvious why senescence pathways would be actively selected in post‐mitotic cells. If cellular senescence is an anti‐cancer strategy, one would not expect to find it in post‐mitotic cells. However, if cellular senescence were a general anti‐damage mechanism it would also be useful for post‐mitotic cells. Immune and stem cells, attracted via a SASP like phenotype, could help to remove damage in tissues with predominantly post‐mitotic cells.

## 6 WHY DO SENESCENT CELLS ACCUMULATE WITH AGE?

The evolution of senescent cells, as laid out in the last section, incorporates important experimental findings, but it does not yet explain why this special cell type should actually accumulate with chronological age. Indeed, as outlined above, there should only be a very low level of senescent cells, representing a dynamic equilibrium between being created during tissue repair/remodeling and being removed by the immune system at the end of the repair process.

However, senescent cells do accumulate with age, causing all the negative effects described earlier. What could be the mechanistic reason for this accumulation? Unfortunately, too few facts are currently known to propose a single, specific mechanism, but instead a few plausible scenarios are possible. In this section, we briefly describe a few different scenarios and investigate the consequences using some simple mathematical “toy” models.

### 6.1 Scenario 1: The immune system deteriorates with time

The most simple explanation for the rise of senescent cells in our context would be to assume that the immune system declines functionally because of the aging process (Aw et al., 2007). In this scenario, the driver for the accumulation of senescent cells (the primary aging process) remains unknown. However, it might be informative to see what consequences we can expect for the dynamics of the accumulation process. For this purpose, we assume that senescent cells (SC) appear at a constant rate "*k*" and are removed via the interaction with the immune system, IM(*t*), with “*d*” being a constant controlling the strength of the interaction. To keep things simple, we assume that the function of the immune system itself simply declines with an exponential term.

$$\frac{d\text{SC}}{\mathrm{dt}}=k‐d\cdot \text{IM}\left(t\right)\cdot \text{SC}\left(t\right)\;\text{with IM}\left(t\right)=e^{‐c\cdot t}$$

**Figure 2:**
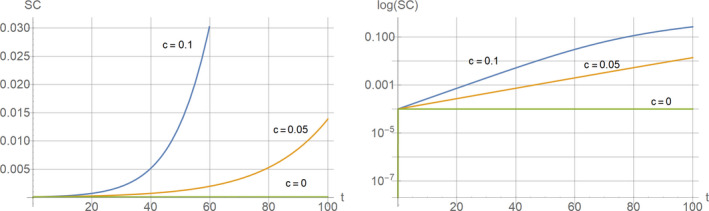
The curves show a typical relationship between time (i.e., organismal age) and the level of senescent cells, SC, under scenario 1. The plot on the left side shows the results on a linear scale, while the right plot displays SC on a logarithmic scale. Parameters used were *k* = 0.01 and *d* = 100 with the shown values for c. For this simple model SC is given in arbitrary units

Figure 2 shows typical simulation results under this scenario for different values of the parameter “*c*” that controls the speed of the decay of the immune system. Not surprisingly, a decline of the immune system leads to a non‐linear accumulation of senescent cells over time. From the logarithmic presentation (Figure 2 right), it is easy to see that this rise is first exponential and later turns into a linear accumulation as IM(*t*) effectively becomes zero. It can also be seen that, only if there is no decline of the immune system (*c* = 0), is a steady state level of SC reached, which is given by *k*/*d* (here 10^−4^).

### 6.2 Scenario 2: SASP converts healthy cells into further senescent cells

Scenario 1 can explain the accumulation of senescent cells with age, but is somewhat unsatisfactory since instead of providing an explanation for the aging process, it simply assumes a deterioration of the immune system without further justification. Here, we explore the possibility that the immune system remains constant, but we now take into account the finding that senescent cells exert a bystander effect that can convert neighboring cells into further senescent cells (Nelson et al., 2012; da Silva et al., 2018; Xu et al., 2017). For this, we modify the equation from scenario 1 to highlight that the immune system now remains constant (IM) and to include a term that describes the creation of senescent cells through existing senescent cells (controlled via parameter “*p*”).

$$\frac{d\mathrm{SC}}{\mathrm{dt}}=k+p∙\mathrm{SC}\left(t\right)‐d∙\mathrm{IM}∙\mathrm{SC}\left(t\right)$$

**Figure 3:**
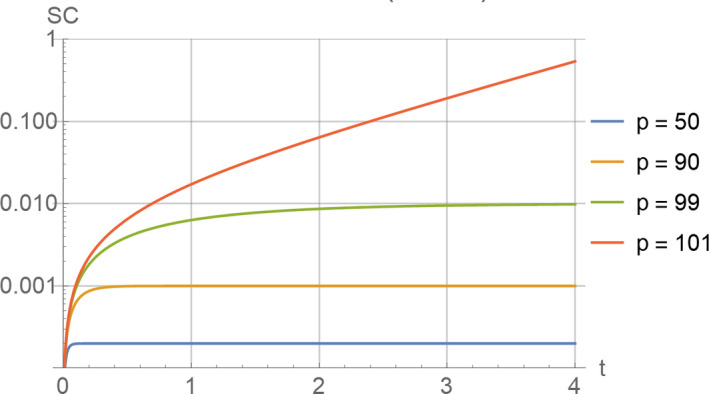
The curves show a typical relationship between time and the level of senescent cells, SC under scenario 2. The plot is drawn on a logarithmic scale and shows the accumulation of SC for various values of the parameter *p*. Other parameters used were IM=1, *k* = 0.01 and *d* = 100

Figure 3 shows typical time courses for SC under this scenario, depending on different values for the parameter “*p*.” It can be shown that, as long as *c* < d * IM, the system will reach a steady state for SC given by *k*/(d * IM‐*c*). If, however, c > *d* * IM, senescent cells accumulate exponentially without limits, as shown in the diagram for *p* = 101.

### 6.3 Scenario 3: The immune system cannot recognize all senescent cells

Although several further scenarios are possible, we want only to concentrate on one more, which seems quite plausible. This last scenario highlights the fact that the immune system needs to recognize senescent cells, before it can remove them. This might be more difficult than it sounds, since so far a marker that could identify senescent cells with perfect specificity is still elusive. SA‐β‐Gal and p16 are currently often used to identify senescent cells (Campisi & d'Adda di Fagagna, 2007; Collins & Sedivy, 2003; Dimri et al., 1995), but they fail to identify all of them, while at the same time cells of the immune system, like macrophages, express p16 without being senescent (Hall et al., 2017). Furthermore, senescent cells are quite heterogeneous regarding their transcriptional profile and SASP composition, depending on the original cell type and type of induction of cellular senescence (Coppe et al., 2010; Hernandez‐Segura et al., 2017, 2018), which complicates a common recognition mechanism even more. Various mechanisms exist by which cells of the immune system recognize senescent cells (Burton & Stolzing, 2018; Kale et al., 2020), and recent observations indicate that senescent cells can actively influence their own clearance. Pereira et al. (2019) showed that senescent cells express the non‐canonical MHC molecule HLA‐E, which interacts with inhibitory receptors on natural killer and CD8 cells, leading to a diminished immune clearance. Additionally, the expression of HLA‐E is influenced in a paracrine fashion by the SASP. Thus, it seems reasonable that senescent cells have in vivo a range of survival times caused by different visibilities for the immune system.

Furthermore, there are probably stringent hurdles for the removal of senescent cells by the immune system, since this amounts to killing the body's own cells. The immune system has to strike a delicate balance between the consequences of false‐positive and false‐negative recognition events for the body. It might be better for the immune system not to kill body cells too aggressively (and risk killing the wrong cells) and instead to allow some senescent cells to remain undetected. Therefore, our last scenario investigates the consequences if there would be a spectrum, such that some senescent cells are rapidly eliminated, while others are hardly removed at all. For mathematical simplicity, we assume that there are just two types of senescent cells. Those that are removed as described above, and a small fraction of senescent cells that are not eliminated by the immune system, because they are not recognized. That means, we have now equations for two types of senescent cells, removable ones, SCr, and non‐removable ones, SCn, as well as a new parameter “a” that specifies, which fraction of newly generated senescent cells belong to SCr.

$$\frac{d\mathrm{SCr}}{\mathrm{dt}}=a∙k+a∙p∙\left(\mathrm{SCr}\left(t\right)+\mathrm{SCn}\left(t\right)\right)‐d∙\mathrm{IM}∙\mathrm{SCr}\left(t\right)$$

$$\frac{d\mathrm{SCn}}{\mathrm{dt}}=\left(1‐a\right)∙k+\left(1‐a\right)∙p∙\left(\mathrm{SCr}\left(t\right)+\mathrm{SCn}\left(t\right)\right)$$

**Figure 4:**
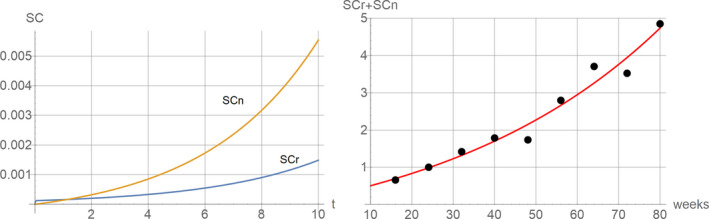
Left: Typical relationship between time and the level of removable and non‐removable senescent cells under scenario 3. Parameters used are IM=1, *k* = 0.01, *p* = 20, *d* = 100 and *a* = 0.99. Right: Sum of SCr and SCn with parameters fitted to longitudinal measurements of senescent cells taken from Burd et al. (2013) and normalized to give a mean abundance of 1 at 24 weeks of age. Best fit parameters: *k* = 0.148, *p* = 0.128, *d* = 0.637 and *a* = 0.883 (with IM = 1)

Figure 4 (left) shows a typical behavior of the model. Since senescent cells generate additional senescent cells via the bystander effect (if *p* > 0), the amount of non‐removable as well as removable SC increases exponentially. The presence of non‐removable SC prevents the system reaching a steady state, in contrast to scenario 2. Recently, Karin et al. (2019) published an interesting study, where they fitted a range of mathematical models to experimental longitudinal measurements of p16 positive cells in mice, taken from Burd et al. (2013). For comparison, we performed a similar fit for our model such that the sum of removable and non‐removable SC approximates the experimental data. As can be seen in Figure 4 (right) our model also achieves an excellent fit, comparable to that shown in Figure 2c of Karin et al. (2019), suggesting that it offers a potential candidate to explain the data, to be clarified in future work. The model of Karin et al. (2019) also predicted a reduced turnover of senescent cells with age, which they verified experimentally using bleomycin‐induced senescent cells in mice lungs. Similarly, our model results in such a decreasing turnover rate since the fraction of removable SC (SCr/(SCr + SCn)) shrinks over time (data not shown). This means that the half‐life of the total population of senescent cells increases with age.

### 6.4 Replicative senescence

As described above, cellular senescence can be caused by several different damaging mechanisms. Replicative senescence, driven by the shortening of telomeres associated with cell division, is only one of these processes. However, it is special since it could be avoided merely by activating telomerase, whereas all other triggers of cellular senescence are based on some form of sporadic damage that cannot be completely avoided. Thus, the question arises: how does replicative senescence fit in the proposed view of the evolution of cellular senescence?

In this respect, it is instructive to see how telomerase is expressed in different species. Telomerase activity has been measured in 15 rodent species and a negative correlation was found with body mass but not with life span (Seluanov et al., 2007). Thus, there are not only short‐lived, but also long‐lived species of rodents, like the naked mole rat and gray squirrel that express telomerase. Similarly, it has also been shown that some long‐lived birds express telomerase (Haussmann et al., 2007). Curiously, while in most farm animals no telomerase activity was found, it has been reported that multiple tissues of pigs express the enzyme (Gorbunova & Seluanov, 2009). This result is noteworthy since pigs do not seem to suffer from higher cancer rates than other farm animals, which suppress telomerase.

If replicative senescence acts as an anti‐cancer mechanism by limiting the number of possible cell divisions, it seems that the actual rate of telomere loss is of greater relevance than raw telomerase activity, since reactive oxygen species and various stressful conditions can influence the rate of shortening (Cerchiara et al., 2017; von Zglinicki et al., 1995). Indeed it has been shown that DNA damage limits the division potential of mouse fibroblasts under standard culture conditions (20% O_2_), while they reach more than 60 population doublings when kept under 3% oxygen (Parrinello et al., 2003), which is in agreement with the fact that mice have long telomeres and express telomerase. Investigating the telomere rates of change in 14 species of birds, it was found that species characterized by longer maximum life spans had a statistically significant slower rates of telomere loss (Dantzer & Fletcher, 2015). Indeed, for some very long‐lived birds and bats no telomere shortening at all could be detected (Cerchiara et al., 2017; Foley et al., 2018). This is the opposite of what would be expected from an anti‐cancer strategy.

So, a possible answer to the question why telomerase is switched off in humans could simply be because it is just one of several possible combinations of telomere length, rate of telomere loss and telomerase activity that allows humans to live to their current life span. It may be that we simply misinterpret the lack of telomerase activity in relation to cancer because it is so deceptive.

## 7 DISCUSSION & CONCLUSIONS

Cellular senescence is a phenomenon that has been known about for a long time. During recent years, it has gained growing interest as its causal involvement in the aging process has been corroborated by several experimental findings. Because of this, several groups and companies are developing senolytic approaches that aim to remove senescent cells from aged animals in the hope of achieving a rejuvenation and life extension effect. However, at the same time, cellular senescence is also seen as an anti‐cancer strategy, which raises the question why interfering with an anti‐cancer mechanism should increase life span?

**Figure 5:**
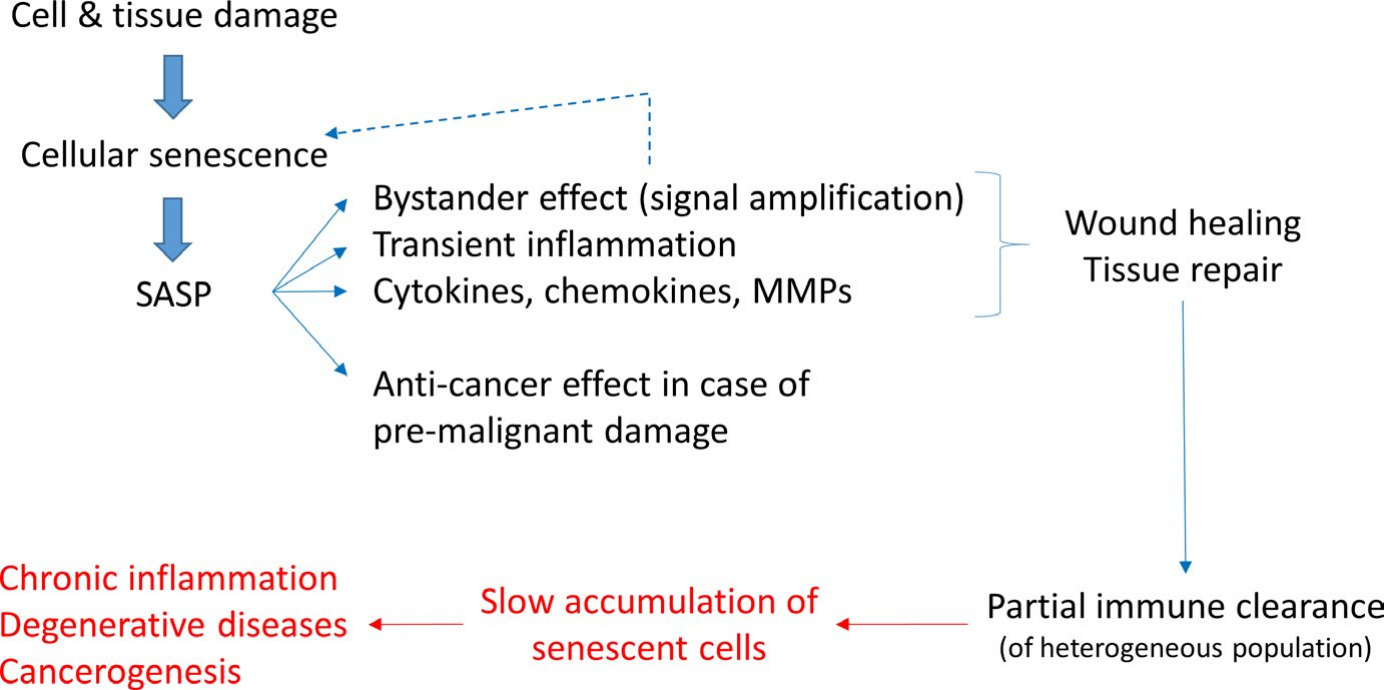
Cellular senescence might have evolved as a tissue repair strategy. In this case, senescent cells were only present temporarily and their SASP would serve a meaningful purpose. According to this proposal, senescent cells would accumulate and cause negative effects (shown in red) because the immune system is not capable of performing a complete immune clearance (see main text for details)

The argument that antagonistic pleiotropy explains the anti‐tumorigenic as well as the pro‐tumorigenic and inflammatory properties of senescent cells is problematic, since there are multiple ways imaginable to break the link between positive and negative effects. In this paper, we discussed an alternative idea for the evolution of cellular senescence that focuses on the involvement of senescent cells in the repair of cell and tissue damage. From such a viewpoint, many properties of the SASP make much more sense and are actually beneficial (Figure 5). Additionally, the recent finding that also post‐mitotic cells can display characteristics of cellular senescence, agrees well with this idea. While post‐mitotic cells benefit from triggering a healing and repair mechanism, they do not profit from an anti‐cancer process.

According to our interpretation, the negative effects of cellular senescence only emerge because the clearance of senescent cells by the immune system, once the repair process has finished, is imperfect (scenario 3). Senescent cells represent a very heterogeneous population, depending on the original cell type and on how senescence was triggered. We therefore proposed in scenario 3 that there is a continuum of turnover rates, since the immune system is more or less capable of recognizing this range of subtypes. The resulting mathematical model, which for simplicity only uses two types of senescent cells (removable and non‐removable), achieves an excellent fit to experimental data, similar to that presented by Karin et al. (2019). Interestingly, although different in structure, our model also predicts a slowdown of senescent cell turnover with age, in our case explained by an accumulation of non‐removable senescent cells relative to removable ones. Furthermore, in our favored model new senescent cells are generated at a fixed rate (*k*) plus a feedback based on the bystander mechanism, while in the model of Karin et al. (2019) the generation depends explicitly on time (*η* * *t*). It is not quite clear which biological mechanism is responsible for the increase with time. A prediction from scenario 3 is that over time the composition of the senescent cell population changes in such a way that it becomes more difficult for the immune system to recognize and remove them. To test this, it would be necessary to isolate senescent cells from animals of different ages and measure their removal after injection into young mice. However, it should be noted that scenario 3 alone is probably too simple to explain the *in vivo* situation completely. The kinetics of the removal of bleomycin‐induced senescent cells, as described by Karin et al. (2019), is likely to also involve a deterioration of the immune system (as discussed under scenario 1).

For obvious reasons, there are high hurdles for the destruction of body cells. We propose that for this reason, the optimal strategy is for the immune system to accept a small fraction of false negatives, leading to the slow accumulation of senescent cells in the body. This, in turn, then leads to life‐threatening consequences like chronic inflammation (inflammaging), degenerative diseases, and cancer (Figure 5). In this interpretation of cellular senescence, there needs to be a balance between beneficial effects (i.e., wound healing and tissue repair) and negative consequences (i.e., accumulation of senescent cells with inflammation and diseases).

To see how this differs from the idea that cellular senescence is an anti‐cancer strategy, we have to return to the questions that we posed earlier. Why did evolution not break the connection of the antagonistic effects and why do organisms not rely exclusively on apoptosis as anti‐cancer strategy? The discussed proposal makes it more difficult to break the antagonistic effects, since there is always a trade‐off between overlooking too many senescent cells (false negatives) and killing too many healthy body cells (false positives). However, the situation can be improved quantitatively by somehow enabling the immune system to better recognize senescent cells. Indeed, it may be that this has already happened during the evolution of long‐lived species, which accumulate senescent cells at a slower pace than short‐lived species. Within this framework, apoptosis represents the main strategy of an organism to combat cancer. However, Figure 5 shows that cellular senescence may provide an additional anti‐cancer effect, if the provoking damage is of a type that would lead the cell on a path toward malignant transformation. It may also be that overactive deployment of apoptosis to protect against cancer could have pro‐aging effects if it needlessly accelerates age‐related loss of tissue cellularity, when simply shutting down a cell's proliferative potential would suffice (Kirkwood, 2002). One such example could be the existence of senescent cells that cannot be replaced after removal, as has been shown in the case of liver sinusoidal epithelial cells by Grosse et al. (2020). Either way, the anti‐cancer protection would only be a (positive) side effect of cellular senescence, since it is proposed that the primary function of cellular senescence is in wound healing and tissue repair.

If this outline of the evolution of cellular senescence is correct, it also follows that the removal of accumulated senescent cells is a good strategy, as long as it does not interfere with the primary function of this process. Thus, a brief senolytic treatment would be suitable, while a chronically administered drug might be problematic. Another reason to avoid chronic administration is connected to the imperfect specificity of current senolytic compounds. Macrophages expressing p16 (Hall et al., 2017) might be targeted by senolytics, which could be detrimental if macrophages are involved in the removal of senescent cells through the immune system. Similarly, treatments that aim at suppressing or neutralizing the SASP (reviewed in Childs et al., 2017) could have negative effects, since according to the presented interpretation the SASP is an important aspect of the tissue repair function of senescent cells. It will be interesting to see if future experimental findings in this exciting field agree with and corroborate this view of the evolution of cellular senescence.

## CONFLICT OF INTEREST

The authors declare that there are no conflicts of interest.

## AUTHOR CONTRIBUTIONS

AK designed the overall concept of the manuscript and developed the mathematical simulations. AK, JFP, and TK were all involved in writing the text, and all authors reviewed and approved the manuscript.
